# Numerical simulations for the Toda lattices Hamiltonian system: Higher order symplectic illustrative perspective

**DOI:** 10.1371/journal.pone.0215054

**Published:** 2019-04-18

**Authors:** Asif Mushtaq, Amna Noreen, Muhammad Asif Farooq

**Affiliations:** 1 Seksjon for matematikk, FLU, Nord Universitet, N-8049 Bodø, Norway; 2 Department of Mathematics, School of Natural Sciences (SNS), National University of Sciences and Technology (NUST), Islamabad 44000, Pakistan; Escuela Superior Politecnica del Litoral, ECUADOR

## Abstract

In this paper we apply some higher order symplectic numerical methods to analyze the dynamics of 3-site Toda lattices (reduced to relative coordinates). We present benchmark numerical simulations that has been generated from the HOMsPY (Higher Order Methods in Python) library. These results provide detailed information of the underlying Hamiltonian system. These numerical simulations reinforce the claim that the symplectic numerical methods are highly accurate qualitatively and quantitatively when applied not only to Hamiltonian of the Toda lattices, but also to other physical models. Excepting exactly integrable models, these symplectic numerical schemes are superior, efficient, energy preserving and suitable for a long time integrations, unlike standard non-symplectic numerical methods which lacks preservation of energy (and other constants of motion, when such exist).

## 1 Introduction

Hamiltonian equations of motion belong to a class of ordinary differential equations (ODEs) which in general are difficult or mostly impossible to solve analytically. Consider a separable Hamiltonian written in the form

$$\begin{array}{c} H\left(q,p\right)=\frac{1}{2}p^{T}Mp+V\left(q\right)=T\left(p\right)+V\left(q\right), \end{array}$$ (1)

where *T*(***p***) is the non-relativistic kinetic energy, *V*(***q***) is the potential energy and *M* is the inverse mass matrix. The autonomous Hamiltonian equations of motion constitute a system of first order ordinary differential equations,

$$\begin{array}{c} {\dot{q}}^{a}=\frac{\partial H}{\partial p_{a}},\;{\dot{p}}_{a}=-\frac{\partial H}{\partial q^{a}},\;a=1,\ldots N \end{array}$$ (2)

where *q*^*a*^ and *p*_*a*_ are generalized coordinates of positions and momenta, respectively. The ” ˙ ” denotes differentiation with respect to time *t*, and ***H*** = *H*(***q***, ***p***). The initial conditions at *t* = 0 can be written as,

$$\begin{array}{c} q^{a}\left(0\right)=q_{0}^{a},\;p_{a}\left(0\right)=p_{a0}. \end{array}$$

We define Eq (2) in abbreviated form as,

$$\begin{array}{c} z=(\begin{array}{c} q\\ p \end{array}),\;\nabla H=(\begin{array}{c} \partial H/\partial q^{a}\\ \partial H/\partial p_{a} \end{array}), \end{array}$$ (3)

$$\begin{array}{c} J=(\begin{array}{cc} 0 & I\\ -I & 0 \end{array}), \end{array}$$ (4)

where ***J*** is a skew-symmetric matrix. Further ***I*** and **0** represent the (N×N) unit and zero matrices, respectively. The compact conservative Hamiltonian system in differential form is,

$$\begin{array}{c} \dot{z}=J^{-1}\nabla H\left(z\right). \end{array}$$ (5)

The above equations of motion are equivalent to Newton’s second law of mechanics with conservative forces. The dynamics generated by these equations define, for each evolution time, a mapping between regions of phase space. A general feature of these mappings is that they preserve the volume of the regions being mapped (and some related properties, collectively referred to as the symplectic structure). The standard non-symplectic numerical integrators, that have been used to solve general initial value problems numerically, do not preserve this qualitative behaviour, or the constants of motion for the system. Examples of such numerical integrators are the classical Runge-Kutta methods of different order, as found in standard integration packages.

By contrast the geometrical numerical integrators have gained popularity in the scientific community, due to their geometry preserving properties, in order to find qualitatively better solutions to Hamiltonian problems. In physical systems energy preservation, symmetries, time-reversal invariance, symplecticity, angular momentum, phase-space volume and dissipation are some key and crucial components to understand geometric properties. Detailed discussions of symplectic integrators with geometric properties have been given in [1–4].

Since the symplectic solvers have been widely accepted to be superior than the conventional numerical methods for solving the Hamiltonian systems, Mushtaq *et. al*. [5] constructed a well behaved class of higher order symplectic integrators schemes based on the extensions of the Störmer-Verlet scheme for Hamiltonians like Eq (1). An overview of these extensions are presented in Section 3. In this paper, we apply these schemes to the Toda lattice models.

The new proposed (*KiMoKi*) schemes involve extensive calculations of higher order derivatives of the Hamiltonian; hence it becomes a nightmare to do correct implementations manually. A collection of Python program *HOMsPy* (Higher Order Methods in Python) has been developed and presented by Mushtaq and Olaussen [8] to overcome these cumbersome and error-prone calculations for higher accuracy. More details, with implementation of many applications, can be found tutorial on HOMsPY by Mushtaq [9].

The structure of the rest of this paper is as follows: In Section 2 we review the Toda lattice models. These are integrable, nonlinear systems that have a number of extra constants of motions beyond standard ones like energy and momentum. The form of these can be described in a very consise manner by Eqs (7) and (8). In Section 3 we review the construction of the *KiMoKi* class of symplectic numerical solvers. In Section 4 we present and discuss the numerical simulations of the Toda lattice models by use of these methods. In Section 5 we conclude the main body of paper with brief remarks. Some technical details are delegated to appendices.

## 2 Toda lattices

The periodic Toda lattice with N sites (or particles) can, in suitable dimensionless coordinates, be specified by the Hamiltonian [10],

$$\begin{array}{c} H\left(q,p\right)=\frac{1}{2}\sum_{a=1}^{N}p_{a}^{2}+\sum_{a=1}^{N}(\exp(q_{a}-q_{a+1})-1), \end{array}$$ (6)

where *q*_*a*_ and *p*_*a*_ are phase-space coordinates of positions and momenta respectively, and the index *a* is interpreted modulo N (i.e., $q_{N+1}\equiv q_{1}$). This mode belongs to a more general class of lattice models where the nearest-neighbour potential, exp(*q*) − 1, is replaced by an arbitrary function *V*(*q*). Another famous member of this class is the Fermi-Pasta-Ulam-Tsingou problem, with *V*(*q*) = *q*^2^/2 − *αq*^3^/3 + *βq*^4^/4. The original study by Fermi *et. al*. [11] only treated linear chains with fixed endpoints, and parameter choices for which *αβ* = 0.

Integrability is one of the most important properties of the Toda lattices. The model describes a set of equal mass particles moving on a ring with exponentially decreasing nearest neighbour interactions. The 2N phase-space coordinates can be used to define a symmetric, periodic tridiagonal N×N (time dependent) matrix,

$$\begin{array}{c} L=(\begin{array}{ccccc} p_{1} & v_{1} & 0 & \cdots & v_{N}\\ v_{1} & p_{2} & v_{2} & \cdots & 0\\ 0 & v_{2} & p_{3} & \cdots & 0\\ \vdots & \vdots & \ddots & \ddots & \vdots \\ v_{N} & 0 & \cdots & v_{N-1} & p_{N} \end{array}), \end{array}$$ (7)

where *v*_*a*_ = −e^(*q*_*a*_−*q*_*a*+1_)/2^. It was shown by Flaschka [12], using theory developed by Lax [13], that the eigenvalues λ_*a*_ of *L* remain unchanged if the evolution *q*_*a*_(*t*), *p*_*a*_(*t*) is generated by the Hamiltonian of Eq (6). This means that all quantities

$$\begin{array}{c} C_{n}\equiv \sum_{a=1}^{N}\lambda_{a}^{n}=\text{Tr}\;L^{n} \end{array}$$ (8)

are constants of motions. The first two are familiar, general expressions,

$$\begin{array}{c} C_{1}=\sum_{a=1}^{N}p_{a}\equiv P\;\left(\text{total}\;\text{momentum}\right), \end{array}$$ (9a)

$$\begin{array}{c} \frac{1}{2}C_{2}=\sum_{a=1}^{N}[\frac{1}{2}p_{a}^{2}+v_{a}^{2}]=H+N\;(\text{total}\;\text{energy}). \end{array}$$ (9b)

The third one is a special consequence of integrability,

$$\begin{array}{c} C_{3}=\sum_{a=1}^{N}[p_{a}^{3}+3p_{a}\left(v_{a-1}^{2}+v_{a}^{2}\right)]+6\delta_{N,3}\;v_{1}v_{2}v_{3}, \end{array}$$ (9c)

where the last term is just an uninteresting constant, since *v*_1_*v*_2_*v*_3_ = 1 when N=3.

It may not be easy to discover a general prescription like the one above. Alternative methods to find additional conservation laws (when one suspects that such exists) are the more *brute force* type of searches used by Göktaş *et. al*. [14] and Hohler *et. al*. [15]. They started by deducing the general form of a possible conservation law, with unknown coefficients, and next tried to explicitly solve for the coefficients with the help of computer algebra. This is a more pedestrian and cumbersome approach, but may be more likely to succeed when applied to models with unknown properties.

### 2.1 The 3-particle case

For N=3 it is simple to introduce center-of-mass and relative coordinates. A common physicists choice is Jacobi coordinates with corresponding conjugate momenta (see Appendix A),

$$\begin{array}{cc} & X=\frac{1}{3}\left(q_{1}+q_{2}+q_{2}\right),\;x_{1}=q_{1}-q_{2},\;x_{2}=\frac{1}{2}(q_{1}+q_{2})-q_{3},\\ & P=p_{1}+p_{2}+p_{3},\;\pi_{1}=\frac{1}{2}(p_{1}-p_{2}),\;\pi_{2}=\frac{1}{3}(p_{1}+p_{2}-2p_{3}). \end{array}$$

This separates the Hamiltonian into center-of-mass and internal contributions, $H=\frac{1}{6}P^{2}+H_{⊥}$, with

$$\begin{array}{c} H_{⊥}\left(x,\pi \right)=\pi_{1}^{2}+\frac{3}{4}\pi_{2}^{2}+v_{1}^{2}+v_{2}^{2}+v_{3}^{2}-3. \end{array}$$ (10a)

In a similar manner we may rewrite $C_{3}=\frac{1}{9}P^{3}+2PH_{⊥}+3\;C_{3⊥}+6$, with

$$\begin{array}{c} C_{3⊥}=\pi_{1}^{2}\pi_{2}-\frac{1}{4}\pi_{2}^{3}-\pi_{1}\left(v_{2}^{2}-v_{3}^{2}\right)+\pi_{2}\left[v_{1}^{2}-\frac{1}{2}\left(v_{2}^{2}+v_{3}^{2}\right)\right]. \end{array}$$ (10b)

A direct evaluation of *dC*_3⊥_/*dt*, using the Hamilton equations generated by *H*_⊥_, confirms that it vanishes. I.e., that *C*_3⊥_ indeed is a constant of motion.

The Hamiltonian of Eq (10a) can be rewritten by a canonical scale transformation,

$$\begin{array}{c} \left(\pi_{1},\pi_{2},x_{1},x_{2}\right)=(-\frac{1}{4}p_{2},\frac{1}{2\sqrt{3}}p_{1},-4q_{2},2\sqrt{3}q_{1}), \end{array}$$

followed by a change of time and mass units (see Appendix B), $t\to \sqrt{3}t$, $m\to \frac{1}{8}m$. This transforms Eq (10a) to the expression used by Lunsford and Ford [16],

$$\begin{array}{c} H_{⊥}\left(q,p\right)=\frac{1}{2}\left(p_{1}^{2}\;+\;p_{2}^{2}\right)+\frac{1}{24}[\text{exp}\left(2q_{2}\;+\;2\sqrt{3}q_{1}\right)+\text{exp}\left(2q_{2}\;-\;2\sqrt{3}q_{1}\right)+\text{exp}\left(-4q_{2}\right)-3]. \end{array}$$ (11)

With the same transformations the conserved quantity of Eq (10b) can be expressed as

$$\begin{array}{c} {\tilde{C}}_{3⊥}\equiv -96\sqrt{3}C_{3⊥}\\ =8(p_{1}^{2}-3p_{2}^{2})p_{1}+(v_{3}^{2}+v_{2}^{2}-2v_{1}^{2})p_{1}+\sqrt{3}(v_{3}^{2}-v_{2}^{2})p_{2}. \end{array}$$ (12)

If the potential in Eq (11) is expanded to third order in the coordinates *q*_1_, *q*_2_, one obtains the Henon-Heiles [17] Hamiltonian

$$\begin{array}{c} H_{\text{H-H}}=\frac{1}{2}(p_{1}^{2}+p_{2}^{2})+\frac{1}{2}(q_{1}^{2}+q_{2}^{2})+q_{1}^{2}q_{2}^{1}-\frac{1}{3}q_{2}^{3}. \end{array}$$

This is the motivation for the form by Lunsford and Ford [16]. The *KiMoKi* solvers have already been implemented for the Henon-Heiles model by Mushtaq [9], and used to detect chaotic and non-chaotic regions of that model. The former occur for oscillations of larger amplitude, for which the higher order terms in the Toda lattice Hamiltonian become of importance. This explains why chaotic behaviour may occur in the Henon-Heiles model, but not in the Toda lattice models (since they are integrable).

## 3 An overview to construct the higher order symplectic scheme

One idea for construction of a symplectic integrator for the evolution generated by a Hamiltonian,

$$\begin{array}{c} H=T(p)+V(q), \end{array}$$

is to replace it with an iterated sequence of short-time evolutions generated by respectively *T*(***p***) (moves, which changes ***q*** without changing ***p***) and *V*(***q***) (kicks, which changes ***p*** without changing ***q***), since each of these are exactly integrable. This is the Störmer-Verlet scheme, which in its symmetrized form has a global error scaling like the timestep squared, *τ*^2^. One way to achieve higher accuracy is by replacing *T* and *V* by *effective* quantities, *T* → *T*_eff_ and *V* → *V*_eff_, in a systematic manner. The effective quantities will depend on the timestep *τ*, and the wanted order *N* of accuracy, *τ*^*N*^. In the *kick-push-move-kick* scheme proposed by Mushtaq *et. al*. [5], *V*_eff_ is still a function of ***q*** only (in addition to *τ*); hence it can still be treated a potential, only slightly changed. Then this is no longer possible for *T*_eff_; it must depend on both ***p*** and ***q***. However, what is really needed is not the infinitesimal generator *T*_eff_(***p***, ***q***; *τ*), but its corresponding, sufficiently accurate, finite (but short) time generator *G*(***q***, ***P***; *τ*). The latter can be constructed in a systematic manner:

$$\begin{array}{c} G\left(q,P\right)=\sum_{k=0}^{N}G_{k}\left(q,P\right)\;\tau^{k}, \end{array}$$ (13)

such that the transformation (***q***, ***p***) → (***Q***, ***P***) is defined by

$$\begin{array}{c} p_{a}=\frac{\partial G}{\partial q^{a}}, \end{array}$$ (14a)

$$\begin{array}{c} Q^{a}=\frac{\partial G}{\partial P_{a}}, \end{array}$$ (14b)

which preserves the symplectic structure exactly, reproduces the time evolution generated by *T*_eff_ to order *τ*^*N*^. Here *Q*^*a*^ is shorten for *q*^*a*^(*t* + *τ*), and *P*_*a*_ shorten for *p*_*a*_(*t* + *τ*). The change in momentum ***p*** (of order *τ*^3^—i.e. a gentle *push*) is then defined through an implicit equation (but one which has turned out to be unproblematic to solve by iteration for all cases tried), while the change in position ***q*** continues to be explicit. Hence, the evolution step generated by *G* consists of a *move*, accompanied with a gentle *push*.

Define *K* such that 2*K* + 2 is the order of the method, where *K* = 1, 2, 3 and *K* = 0 corresponds to the Störmer-Verlet scheme. One full time step with this modification for *kick-push-move-kick* scheme is,

1. “Kick”:
  $$\begin{array}{c} q^{a}\to q^{a}, \end{array}$$ (15a)
  $$\begin{array}{c} p_{a}\to p_{a}-\frac{\tau }{2}\;\frac{\partial }{\partial q^{a}}\sum_{k=0}^{K}V_{2k}\left(q\right), \end{array}$$ (15b)
2. “Push”:
  $$\begin{array}{c} q^{a}\to q^{a}, \end{array}$$ (15c)
  $$\begin{array}{c} P_{a}=\frac{\partial }{\partial q^{a}}\sum_{k=0}^{2K+2}\tau^{k}\;G_{k}\left(q,P\right). \end{array}$$ (15d)
  But *P*_*a*_ is unknown yet. We have to solve a nonlinear equation to find *P*_*a*_. However our generating function takes the form,
  $$\begin{array}{c} G\left(q,P\right)=q^{a}\;P_{a}+\frac{1}{2}P_{a}^{2}\;\tau +\sum_{k=3}^{2K+2}\tau^{k}\;G_{k}\left(q,P\right). \end{array}$$ (15e)
  Hence Eq (14a) can be written as,
  $$\begin{array}{c} p_{a}=P_{a}+\frac{\partial }{\partial q^{a}}\sum_{k=3}^{2K+2}\tau^{k}\;G_{k}\left(q,P\right) \end{array}$$
  or in a form suitable for an iterative solution,
  $$\begin{array}{c} P_{a}=p_{a}-\frac{\partial }{\partial q^{a}}\sum_{k=3}^{2K+2}\tau^{k}\;G_{k}\left(q,P\right) \end{array}$$
3. “Move”:
  $$\begin{array}{c} q^{a}\to Q^{a}=\frac{\partial }{\partial P_{a}}\sum_{k=0}^{2K+2}\tau^{k}\;G_{k}\left(q,P\right), \end{array}$$ (15f)
  $$\begin{array}{c} P_{a}\to P_{a}. \end{array}$$ (15g)
4. “Kick”:
  $$\begin{array}{c} q^{a}\to q^{a}, \end{array}$$ (15h)
  $$\begin{array}{c} p_{a}\to p_{a}-\frac{\tau }{2}\;\frac{\partial }{\partial q^{a}}\sum_{k=0}^{K}V_{2k}\left(q\right). \end{array}$$ (15i)

The explicit expressions for *V*_2*k*_ and *G*_*k*_ were published by Mushtaq *et. al*. in ref [5]. For convenience, on request from a reviewer, they are included in Appendix D.

To implement these higher order methods for our Toda lattice Hamiltonian, we define the model by the code in Listing 1 of Appendix C, and use programs in the HOMsPy package to automatically generate all the KiMoKi solver code. The complete code package is available as “Supporting information” for this paper.

## 4 Numerical simulations with HOMsPy

As has been mentioned before, numerical simulations on several Hamiltonian systems with the algorithm outlined by Eq (15) has compared favourable to conventional non-symplectic methods. We here present the results of additional simulations, of the model defined by Eq (11), which strengthen this evidence further.

As mentioned earlier that we implemented these numerical schemes as Python routines in HOMsPy. Python is an open source programming language which has gained increasing popularity in general, including (successful) applications for scientific computing. It is fast and easy to code and use for small “prototyping” tasks, since there is no need for explicit declaration of variables or a separate compilation cycle. It also comes with a huge repository of packages covering a large area of applications. Python is equipped with other features which facilitates development and encourages documentation of large well-structured program systems. Obviously, as an interpreted language, native Python is not suitable for performing extended numerical computations. But very often the code for such computations reduces to calls to pre-compiled library routines.

### 4.1 Preservation of constants of motion

We have already stressed the advantage of using symplectic solvers for numerical analysis of Hamiltonian models. This is most important when simulating long time series, where conventional numerical algorithms (like the Runge-Kutta methods usually implemented in numerical packages) can lead to a continuous degradation of important qualitative properties of Hamiltonian systems, like symplecticity (preservation of phase space volume and related quantities) and constants of motion. These methods have no built-in mechanisms for preserving such properties, as is illustrated in this subsection.

As the name suggests, symplectic solvers preserve symplecticity *exactly*. There will, of course, always be errors caused by numerical roundoff, but such errors do not depend on the accuracy of the method, only on the numerical precision being used. Symplectic solvers do not preserve most other constants of motion exactly, but the error (deviation from the initial value) will oscillate in a narrow band around zero. The width of this band scales with the accuracy of the method (i.e., order and timestep) in the expected way. For the symplectic (*KiMoKi*) algorithms used in this paper, applicable to Hamiltonians of the form *H* = *T*(***p***) + *V*(***q***), a constant of motion is preserved exactly if it is conserved separately by *T* and *V*. In this paper, one such example is the total momentum *P* of Eq (9a), while *H* of Eq (9b) and *C*_3_ Eq (9c) are not.

It has been proven that symplectic integrators that preserve the Hamiltonian must actually be exact solvers (modulo errors introduced by finite numerical precision). There exist special methods for integrable models, as f.i. discussed by Kuznetsov and Vanhaecke [6] and Zullo [7]. The KiMoKi integrators, aimed for a more general class of problems, are not able to provide exact solutions, at least not when the conventional coordinates are used.

In this subsection we compare the *KiMoKi* solvers of order 2 and 4 with the RK23 (order 2) and RK45 (order 4) Runge-Kutta methods available through the solve_ivp routine in the scipy.integrate package, for the same values of the timestep *τ* (for the Runge-Kutta solvers, *τ* is the *maximum* timestep).

There are additional methods available in solve_ivp, but they are—for this comparison—inferior to the Runge-Kutta ones.

**Fig 1:**
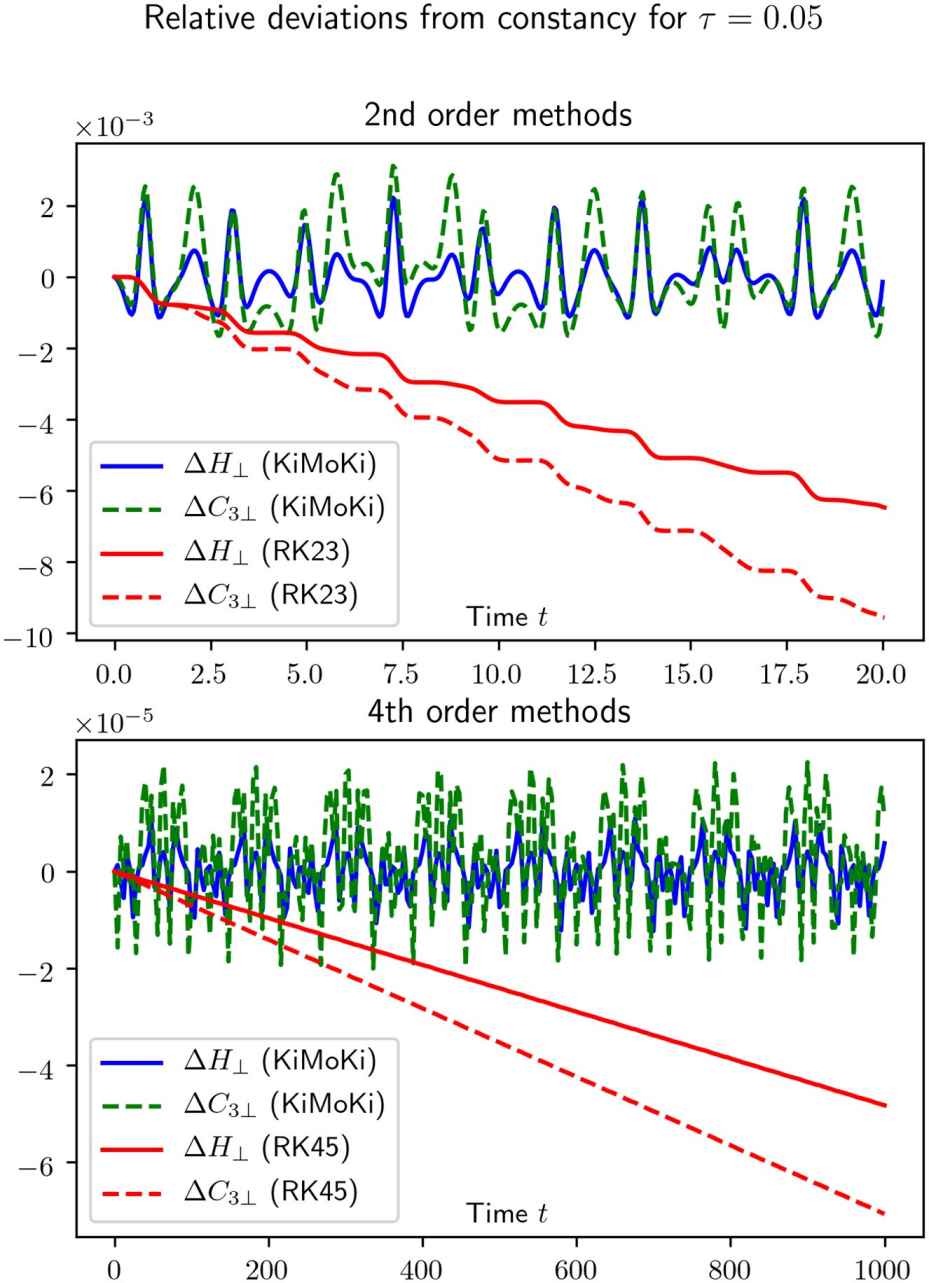
This figure illustrates how well exactly conserved quantities are preserved by our symplectic numerical solvers, compared to the standard Runge-Kutta methods implemented in scipy.integrate.solve_ivp. For the 3-site Toda lattice, reduced to relative coordinates, the constants of motion are the Hamiltonian *H*_⊥_ of Eq (11) and $C_{3⊥}$ of Eq (12). Here Δ*H*_⊥_ = [*H*_⊥_(*t*) − *H*_⊥_(0)]/*H*_⊥_(0), calculated from the numerical solutions, and similar for Δ*C*_3⊥_. Here *τ* is the fixed timestep of the sympletic solvers.

**Fig 2:**
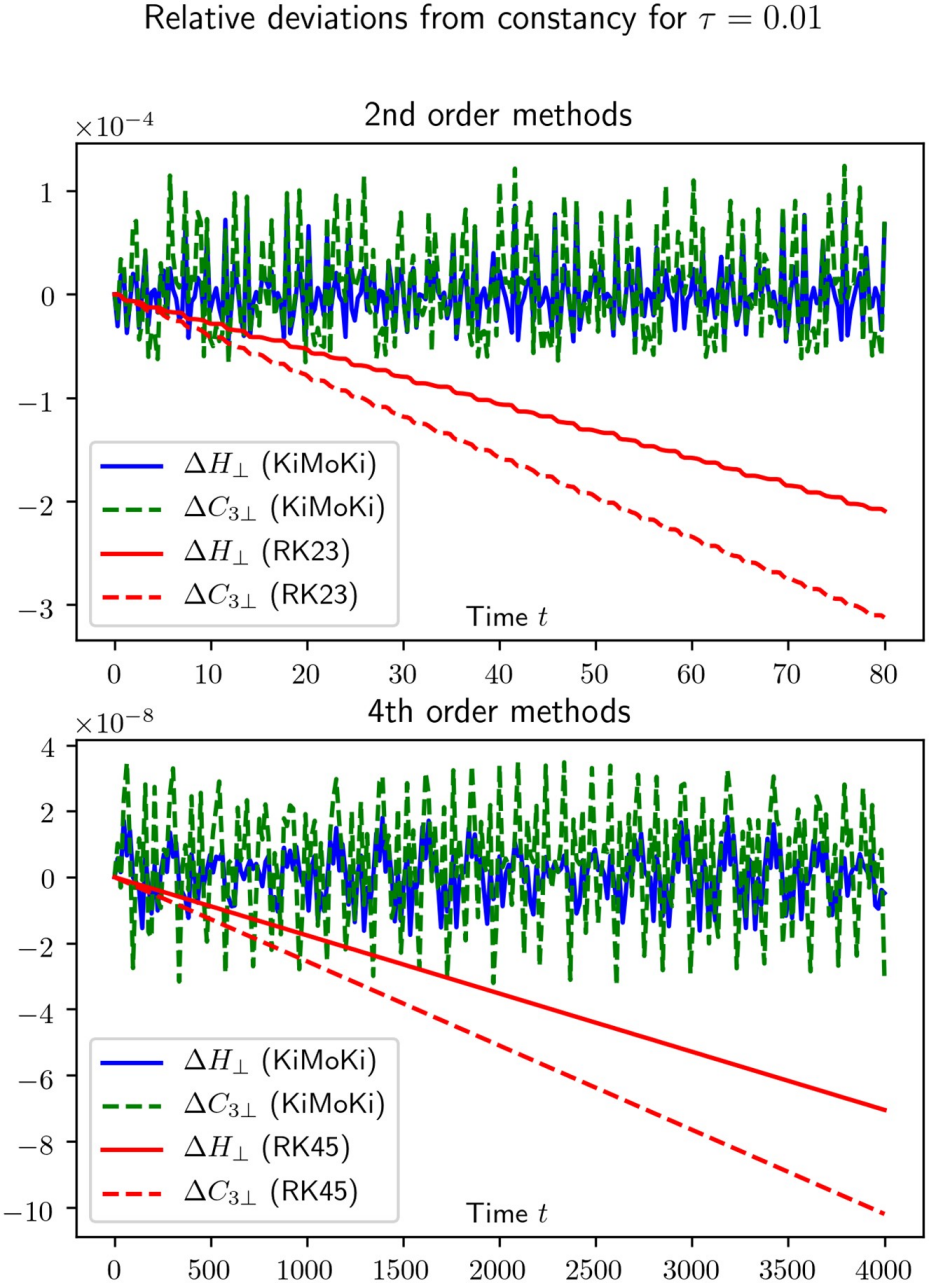
See the caption to Fig 1.

As can be seen from Figs 1 and 2, for short times the Runge-Kutta accuracy may very well be better than the *KiMoKi* ones, but as time increases it steadily becomes worse. By comparing Figs 1 and 2, we note that the time interval in which the Runge-Kutta methods are competitive becomes larger with decreasing *τ*.

### 4.2 Poincaré section technique (surface-of-section)

The reduced 3-site Toda lattice model we investigate has 4 degrees of freedom, *z* ≡ (*q*_1_, *q*_2_, *p*_1_, *p*_2_). Even in this rather simple case it is a challenge to present and visualize how the solutions behave. One, quite popular and efficient method, is the Poincaré section technique (also known as surface-of-section), introduced by Henri Poincaré the early 20th century. Generally, for cases where energy is conserved, *H*(*q*_1_, *q*_2_, *p*_1_, *p*_2_) = *H*_0_, each orbit is restricted to a 3-dimensional constant energy surface of 4-dimensional phase space. The points where one coordinate is kept fixed (for example *q*_2_ = 0) define another, in general independent, 3-dimensional surface. The intersection of these two surfaces is therefore two-dimensional. It can be specified by two coordinates, for example (*q*_1_, *p*_1_). In this example, the points (*q*_1_(*t*_*n*_), *p*_1_(*t*_*n*_)) where the constant energy orbit crosses the *q*_2_ = 0 surface are therefore easy to visualize in two-dimensional plots. The times *t*_*n*_ of crossings, and the order of repeated crossings, will be lost (or visualized by other means).

Repeated crossings will generate a pattern which indicates the nature of the dynamics. In our case, where there is an additional constant of motion (*C*_3⊥_), repeated crossings will appear on two smooth curves—one for each direction in which the (*q*_2_ = 0)-plane is crossed, determined by the initial conditions. For ergodic motion, the crossings should spread smoothly over or more regions of the plane, according to density predicted by classical statistical mechanics. According to KAM theory, perturbations of integrable models are expected to lie in-between: For a finite fraction of initial condition, the crossings will appear on a smooth curve, while the rest will appear to be spread over a region of finite area.

Cheb-Terrab and de Oliveira [18] have written a MapleV R.3 routine for visualizing Hamiltonian dynamics by the Poincaré section technique. They employ the Toda lattice model for usage demonstration. We have implemented their algorithm in python, in combination with the *KiMoKi* solvers. The algorithm uses linear interpolation to determine crossings; hence it is of limited accuracy and is best used with short time-steps *τ*. (All our code could have been implemented in Maple, but this framework is not freely available to all.)

**Fig 3:**
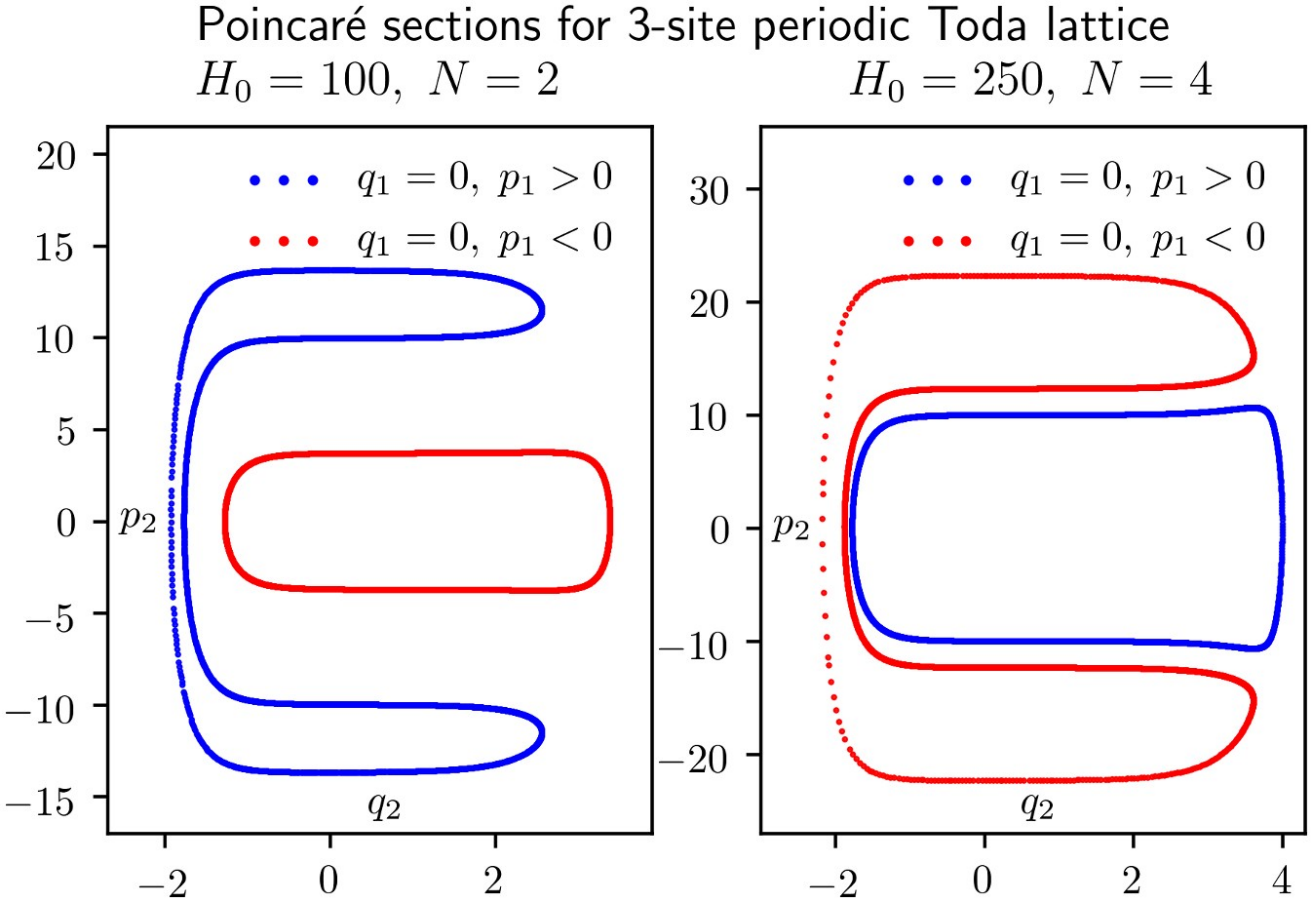
Poincaré sections for an orbit of the reduced 3-site Toda lattice model of Eq (11). Each panel shows 4000 crossings of the (*q*_1_ = 0)-plane, 2000 in the positive direction (*p*_1_ > 0, marked blue), and 2000 in the negative direction (*p*_1_ < 0, marked red). The dynamics between each crossing is determined by *KiMoKi* solvers of order 2 (left panel) resp. 4 (right panel), with timestep *τ* = 0.005. The initial condition is *z*_0_ = (0, 1, 9.95073, 10), with *H*_0_ = 100, *C*_3⊥_ = −15852.72982, for the left panel, and *z*_0_ = (0, 1, 19.97541, 10), with *H*_0_ = 250, *C*_3⊥_ = 16117.70199, for the right panel.

Fig 3 shows 4000 crossings of the orbit with the (*q*_1_ = 0)-plane, 2000 in each direction, using *KiMoKi* solvers of order 2 (left panel) and 4 (right panel) with timestep *τ* = 0.005. For the left panel the initial condition is *z*_0_ = (0, 1, 9.95, 10). The corresponding constants of motions are *H*_0_ = 100, *C*_3⊥_ = −15852.7. For the right panel the initial condition is *z*_0_ = (0, 1, 19.98, 10). The corresponding constants of motion are *H*_0_ = 250, *C*_3⊥_ = 16117.7.

**Fig 4:**
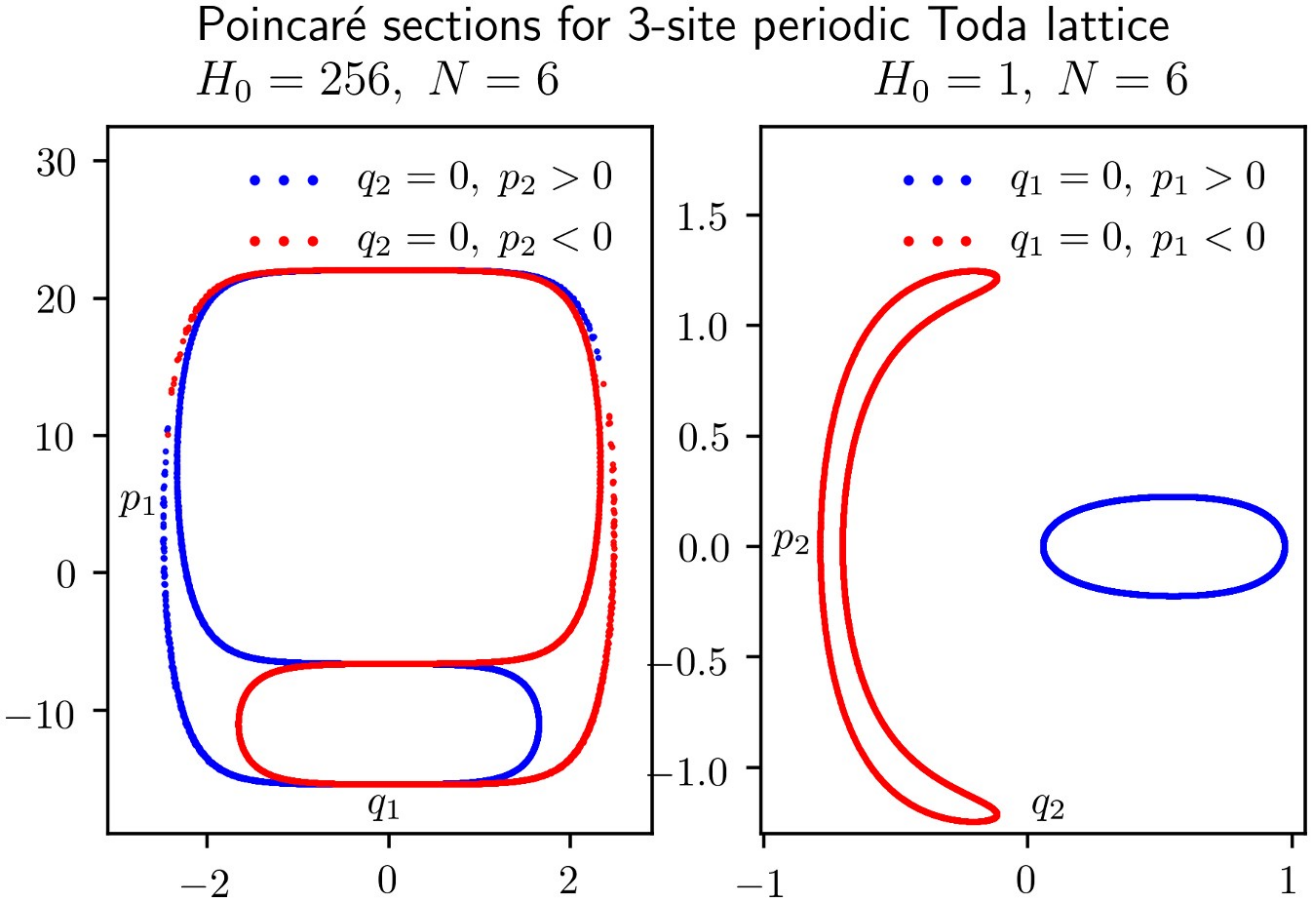
Poincaré sections for an orbit of the reduced 3-site Toda lattice model of Eq (11). Each panel shows 4000 crossings of the (*q*_2_ = 0)-plane, 2000 in the positive direction (*p*_2_ > 0, marked blue), and 2000 in the negative direction (*p*_2_ < 0, marked red). The dynamics between each crossing is determined by a *KiMoKi* solver of order 6, with timestep *τ* = 0.01. The initial condition is *z*_0_ = (1, 0, 22, 5.04993), with *H*_0_ = 256, *C*_3⊥_ = 72099.45264, for the left panel, and *z*_0_ = (0, 0.1, 1.40733), with *H*_0_ = 1, *C*_3⊥_ = 23.51188 for the right panel.

Fig 4 shows 4000 crossings of the orbit with the (*q*_2_ = 0)-plane, 2000 in each direction, using a *KiMoKi* solver of order 6 with timestep *τ* = 0.01. For the left panel the initial condition is *z*_0_ = (1, 0, 22, 5.05). The corresponding constants of motion are *H*_0_ = 256, *C*_3⊥_ = 72099.45. For the right panel *z*_0_ = (0, 0.1, 1.41, 0.1). The corresponding constants of motion are *H*_0_ = 1, *C*_3⊥_ = 23.51.

**Fig 5:**
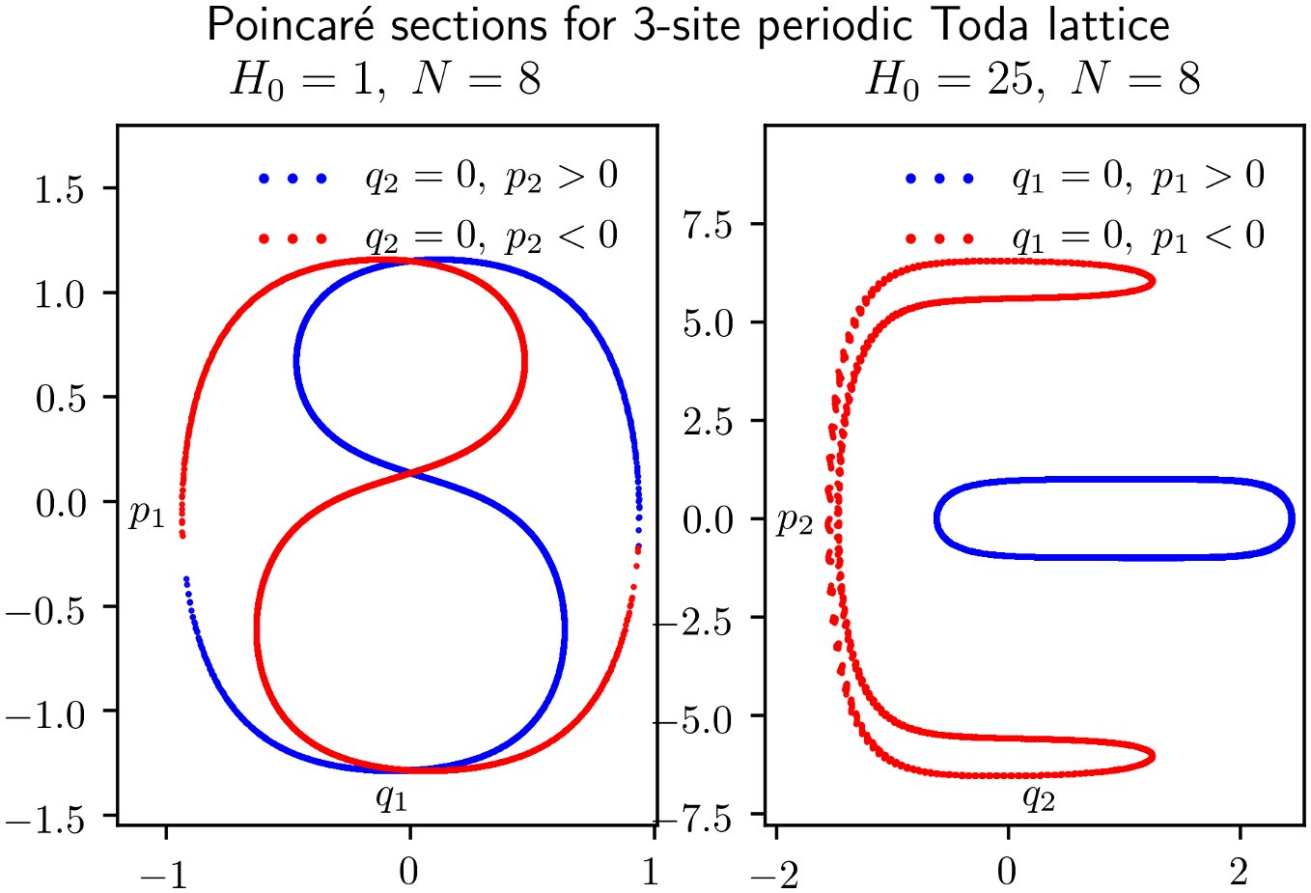
Poincaré sections for an orbit of the reduced 3-site Toda lattice model of Eq (11). The left panel shows 4 000 crossings of the (*q*_2_ = 0)-plane, 2 000 in the positive direction (*p*_2_ > 0, marked blue) and 2 000 in the negative direction (*p*_2_ < 0, marked red). The right panel similarly shows 4 000 crossings of the (*q*_1_ = 0)-plane. The dynamics between each crossing is determined by a *KiMoKi* solver of order 8, with timestep *τ* = 0.05. The initial condition is *z*_0_ = (0.1, 0, 0.1, 1.40709), with *H*_0_ = 1, *C*_3⊥_ = −6.45412, for the left panel, and *z*_0_ = (0, 1, 6.92943, 1), with *H*_0_ = 25, *C*_3⊥_ = 2597.68431, for the right panel.

Fig 5 shows 4 000 crossings of the orbit with the (*q*_2_ = 0)-plane (left panel) or the (*q*_1_ = 0)-plane (right panel), 2 000 in each direction, using a *KiMoKi* solver of order 8 with timestep *τ* = 0.05. For the left panel the initial condition is *z*_0_ = (0.1, 1, 0.1, 1.41). The corresponding constants of motion are *H*_0_ = 1, *C*_3⊥_ = −6.45. For the right panel *z*_0_ = (0, 1, 6.93, 1). The corresponding constants of motion are *H*_0_ = 25, *C*_3⊥_ = 2597.68.

### 4.3 3D camera plots of each orbit

**Fig 6:**
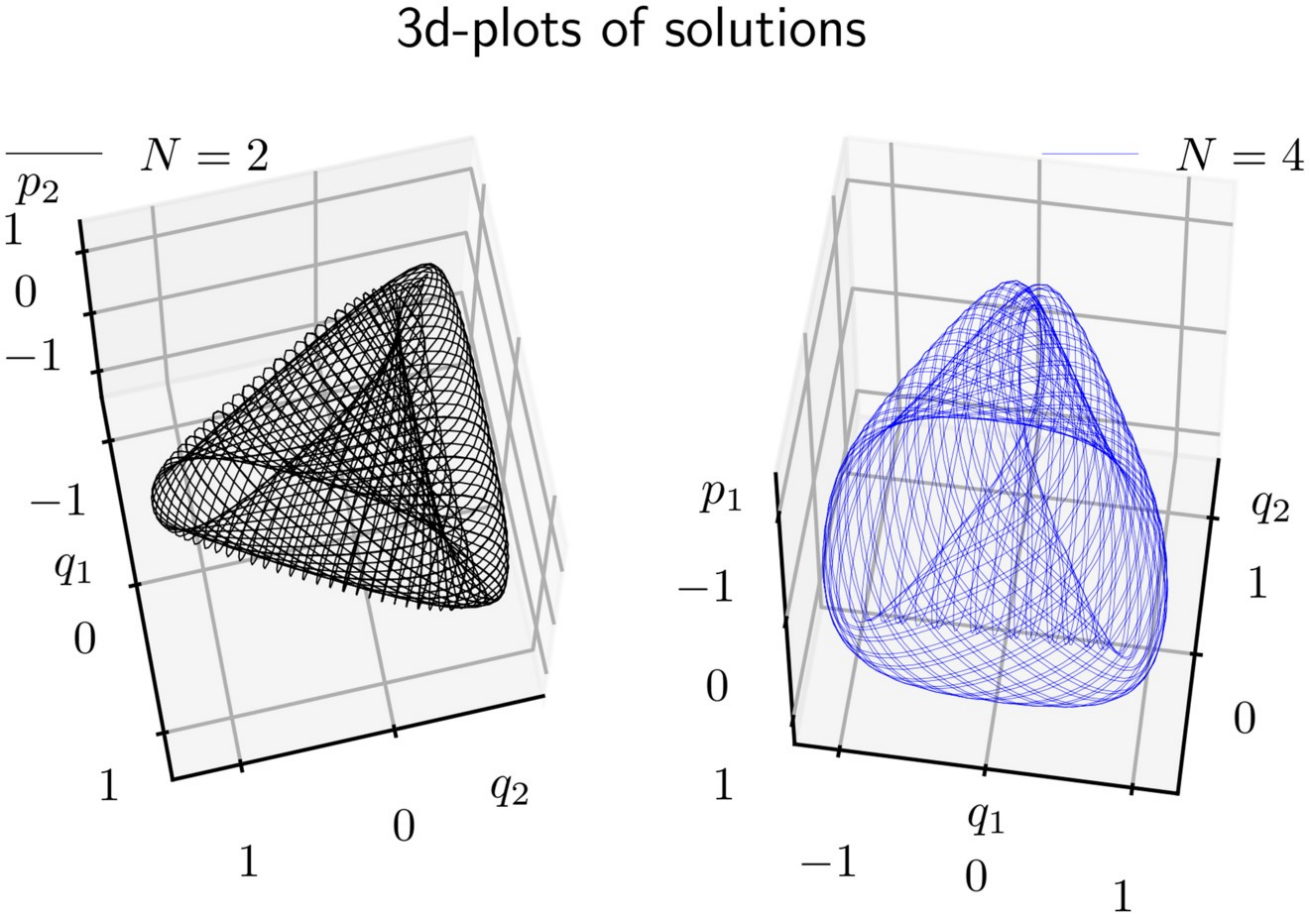
A quasi-periodic orbit *z*(*t*) for times 0 ≤ *t* ≤ 2000, found by the *KiMoKi* solvers of order *N* = 2 (left panel) and order *N* = 4 (right panel) with timestep *τ* = 0.1, projected to respectively the (*q*_1_, *q*_2_, *p*_2_) (left) and (*q*_1_, *q*_2_, *p*_1_) (right) subspaces. The initial condition *z*_0_ = (0.1, 0, 0.1, 1.40709), with *H*_0_ = 1, *C*_3⊥_ = −6.45412. The viewing angle is set to (*ϑ*, *φ*) = (68, 78) (left), respectively (*ϑ*, *φ*) = (−128, −8). Here *ϑ* is the elevation angle (elev) and *φ* the azimuth angle (azim).

**Fig 7:**
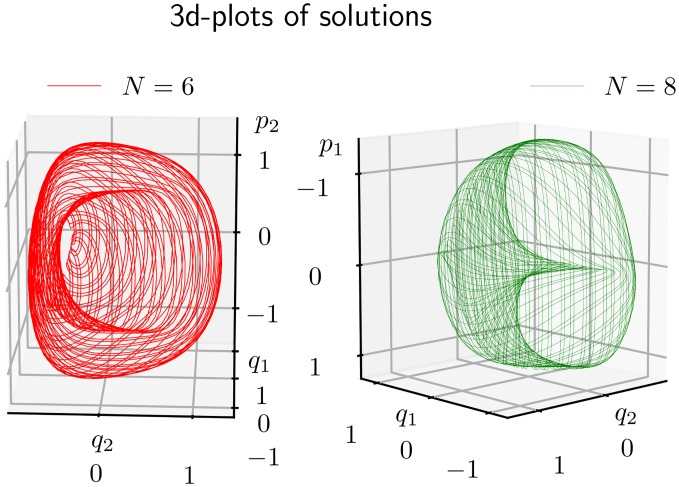
A quasi-periodic orbit *z*(*t*) for times 0 ≤ *t* ≤ 2000, found by the *KiMoKi* solvers of order *N* = 6 (left panel) and order *N* = 8 (right panel) with timestep *τ* = 0.1, projected to respectively the (*q*_1_, *q*_2_, *p*_2_) (left) and (*q*_1_, *q*_2_, *p*_1_) (right) subspaces. The initial condition *z*_0_ = (0.1, 0, 0.1, 1.40709), with *H*_0_ = 1, *C*_3⊥_ = −6.45412. The viewing angle is set to (*ϑ*, *φ*) = (15, −87) (left), respectively (*ϑ*, *φ*) = (−128, 133). Here *ϑ* is the elevation angle (elev) and *φ* the azimuth angle (azim).

An alternative method to visualize the solution behaviour is to make a projection to a 3-dimensional subspace, and display the orbit in a “3-dimensional” plot. This is best done in interactive sessions, since this allows one to vary the viewing direction over all possible spherical angles. Snapshots examples from such matplotlib sessions are shown in Figs 6 and 7, for phase space orbits {*z*(*t*)|0 ≤ *t* ≤ 2 000}. Each plot displays quasi-periodic motion on a two-dimensional surface determined by the initial value *z*_0_ (or more precisely the corresponding constants of motion, *H*_0_ and *C*_3⊥_).

### 4.4 Behavior of energy error

**Fig 8:**
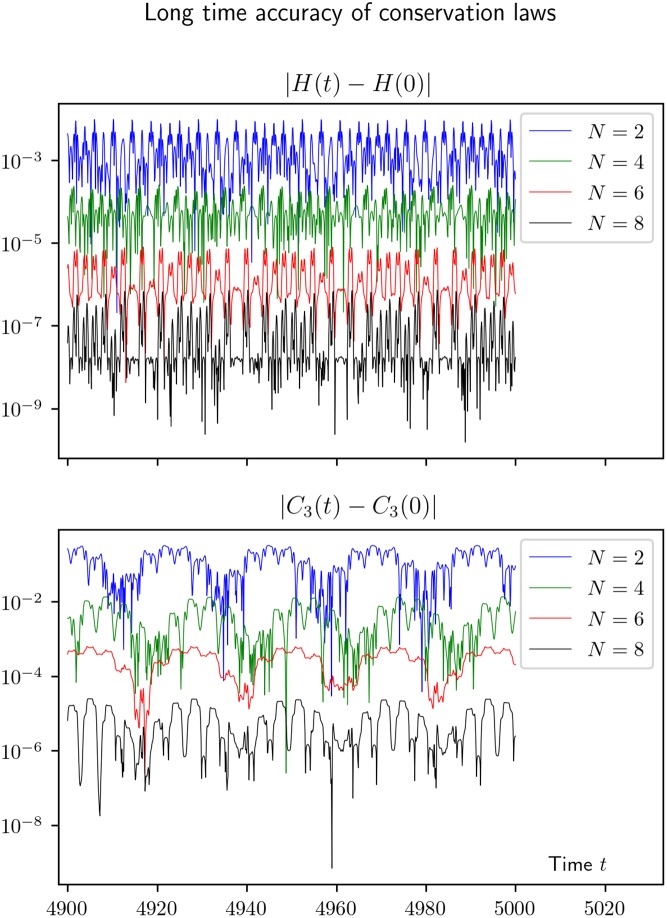
Long time energy error for solutions of a Toda lattice model computed by the *KiMoKi* solvers. An orbit *z*(*t*) with initial value *z*_0_ = (0.1, 0, 0.1, 1.40709), corrsponding to *H*_0_ = 1 and *C*_3⊥_ = −6.45412. The solution is computed for times 0 ≤ *t* ≤ 5 000 with timestep *τ* = 0.1; for better visibility only the last hundred time units are plotted.

We have earlier in Section 4.1 and Figs 1 and 2 shown that the long time behaviour of the *KiMoKi* solvers are better than the standard Runge-Kutta solvers of the same order, with respect to preservation of constants of motion. In Fig 8 we show that this behaviour can be observed for all orders *N* of the *KiMoKi* solvers, with the accuracy increasing with *N* for a fixed timestep *τ*. As can be seen, the errors keep varying in an oscillating manner, with no noticeable increase in amplitude with time.

**Fig 9:**
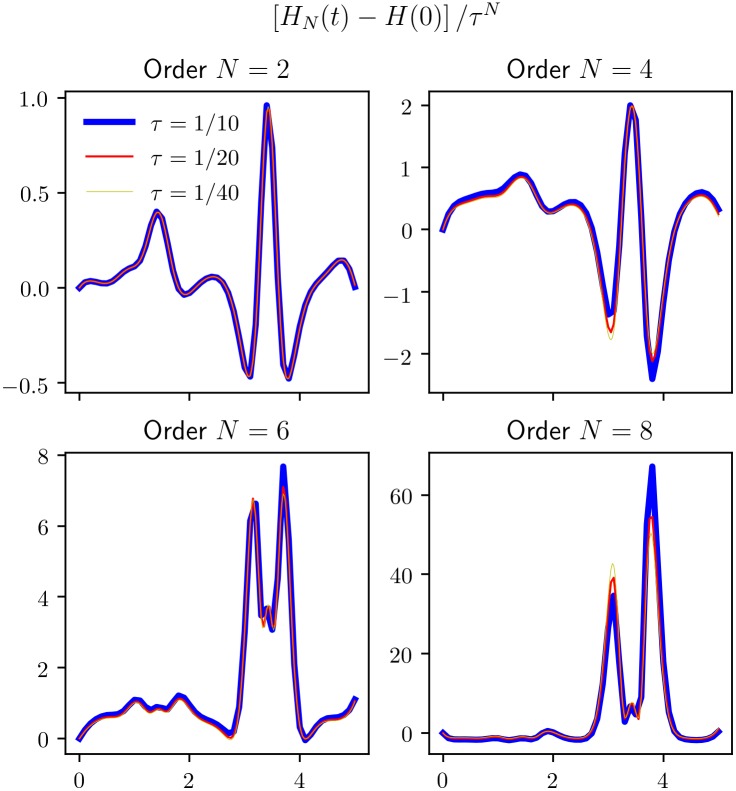
Scaled energy errors for some higher order symplectic integrators, when applied to the reduced 3-site Toda lattice Hamiltonian of Eq (11). The plots are for an orbit *z*(*t*) with initial value (0.1, 0, 0.1, 1.40709), corresponding to *H*_0_ = 1, *C*_3⊥_ = −6.45412, computed with *KiMoKi* solvers of orders *N* = 2, 4, 6, 8, and timesteps *τ* = 0.025, 0.05, and 0.1.

In Fig 9 we further show that the error scales with order *N* and timestep *τ* in the expected manner. I.e., proportional with *τ*^*N*^, with a *N*-dependent constant of proportionality.

## 5 Concluding remarks

In this paper, the *KiMoKi* algorithms for numerical solutions of the Hamilton equations for a Toda lattice model have been discussed and tested. These methods preserve the symplectic structure exactly (within the accuracy given by the employed numerical precision); For order *N* = 2 the method is equal to the Störmer-Verlet scheme, with long-time accuracy of order *τ*^2^; it has been extended to methods of order *τ*^4^, *τ*^6^ and *τ*^8^. As demonstrated, the method works as expected (sometimes even better than expected) for the reduced 3-site Toda lattice model.

A brief summary of this work is as follows:

- The symplectic property is preserved provided we solve the non-linear Eq (14a) for *push* steps to sufficient accuracy.
- Without prior knowledge the quasi-periodic nature of the solutions can easily be detected from 3D plots of the orbits (projected to 3-dimensional subspaces). Further (but not independent) confirmation can be found by investigating the behaviour of the Poincaré section of each orbit.
- Although the *KiMoKi* solver do not preserve constants of motion exactly, the time oscillating error in these quantities do not systematically increase in “amplitude” with time. This amplitude can be reduced in a predictable manner by increasing the order *N* of the method, or decreasing the timestep *τ*, or both.

## A Jacobi coordinates for few-body systems

Consider first a translation invariant Hamiltonian system with two non-relativistic particles of mass *m*_1_ resp. *m*_2_, with position coordinates ***q***_1_ resp. ***q***_2_. To exploit translation invariance it is common to introduce center-of-mass and relative coordinates,

$$\begin{array}{c} (\begin{array}{c} X\\ x \end{array})=(\begin{array}{cc} \mu_{1} & \mu_{2}\\ 1 & -1 \end{array})(\begin{array}{c} q_{1}\\ q_{2} \end{array}), \end{array}$$ (16a)

where *μ*_*j*_ = *m*_*j*_ /(*m*_1_ + *m*_2_), for *j* = 1, 2. For common systems with conjugate momenta $p_{1}=m_{1}{\dot{q}}_{1}$ and $p_{2}=m_{2}{\dot{q}}_{2}$, the new momenta become

$$\begin{array}{c} (\begin{array}{c} P\\ \pi \end{array})=(\begin{array}{cc} 1 & 1\\ \mu_{2} & -\mu_{1} \end{array})(\begin{array}{c} p_{1}\\ p_{2} \end{array}). \end{array}$$ (16b)

The linear transformation of Eq (16) is canonical (because the matrices *M*_***q***_ in Eq (16a) and *M*_***p***_ in Eq (16b) are related by $M_{p}^{t}=M_{q}^{-1}$) and maintains the diagonal form of the kinetic energy. In the equal-mass case, $\mu_{1}=\mu_{2}=\frac{1}{2}$, the matrices *M*_***q***_ and *M*_***p***_ do not become orthogonal. The latter, which can be obtained by scale transformations of ***X*** and ***x***, may look simpler and more natural from a mathematical point of view. However, this would obscure physical interpretation of the coordinates.

The inverse of Eq (16) is

$$\begin{array}{cc} \left(\begin{array}{c} q_{1}\\ q_{2} \end{array}\right) & =(\begin{array}{cc} 1 & \mu_{2}\\ 1 & -\mu_{1} \end{array})(\begin{array}{c} X\\ x \end{array}), \end{array}$$ (17a)

$$\begin{array}{cc} \left(\begin{array}{c} p_{1}\\ p_{2} \end{array}\right) & =(\begin{array}{cc} \mu_{1} & 1\\ \mu_{2} & -1 \end{array})(\begin{array}{c} P\\ \pi \end{array}). \end{array}$$ (17b)

The extension to three particles is obvious for the center-of-mass coordinate, and one may further maintain the previous definition of one relative coordinate. As a second relative coordinate, select the distance between the center-of mass of the first two particles, and the third one. Hence

$$\begin{array}{c} (\begin{array}{c} X\\ x_{1}\\ x_{2} \end{array})=(\begin{array}{ccc} \mu_{1} & \mu_{2} & \mu_{3}\\ 1 & -1 & 0\\ {\tilde{\mu }}_{1} & {\tilde{\mu }}_{2} & -1 \end{array})(\begin{array}{c} q_{1}\\ q_{2}\\ q_{3} \end{array}), \end{array}$$ (18a)

where now *μ*_*j*_ = *m*_*j*_/(*m*_1_ + *m*_2_ + *m*_3_) for *j* = 1, 2, 3 and ${\tilde{\mu }}_{j}=m_{j}/(m_{1}+m_{2})$ for *j* = 1, 2. The new conjugate momenta becomes respectively

$$\begin{array}{c} \left(\begin{array}{c} P\\ \pi_{1}\\ \pi_{2} \end{array})=(\begin{array}{ccc} 1 & 1 & 1\\ {\tilde{\mu }}_{2} & -{\tilde{\mu }}_{1} & 0\\ \mu_{3} & \mu_{3} & -\mu_{1}-\mu_{2} \end{array})(\begin{array}{c} p_{1}\\ p_{2}\\ p_{3} \end{array}\right) \end{array}$$ (18b)

The inverse of Eq (18) is

$$\begin{array}{cc} \left(\begin{array}{c} q_{1}\\ q_{2}\\ q_{3} \end{array}\right) & =(\begin{array}{ccc} 1 & {\tilde{\mu }}_{2} & \mu_{3}\\ 1 & -{\tilde{\mu }}_{1} & \mu_{3}\\ 1 & 0 & -\mu_{1}-\mu_{2} \end{array})(\begin{array}{c} X\\ x_{1}\\ x_{2} \end{array}), \end{array}$$ (19a)

$$\begin{array}{cc} \left(\begin{array}{c} p_{1}\\ p_{2}\\ p_{3} \end{array}\right) & =(\begin{array}{ccc} \mu_{1} & 1 & {\tilde{\mu }}_{1}\\ \mu_{2} & -1 & {\tilde{\mu }}_{2}\\ \mu_{3} & 0 & -1 \end{array})(\begin{array}{c} P\\ \pi_{1}\\ \pi_{2} \end{array}). \end{array}$$ (19b)

For the case of equal masses, $\mu_{j}=\frac{1}{3}$ and ${\tilde{\mu }}_{j}=\frac{1}{2}$.

## B Unit transformations

Most quantities in physical expressions, like the Hamiltonian

$$\begin{array}{c} H=\frac{1}{2m}p^{2}+V\left(q\right), \end{array}$$

are dimensionful. I.e., they carry units of time, length, and mass. When expressed in dimensionless form like in Eq (6) or Eq (10a), this means that the dimensionless time *t*, length *ℓ*, and mass *m* actually are expressed in terms of some reference quantities *t*_0_, *ℓ*_0_, *m*_0_. I.e., a dimensionless potential energy *V*(*q*) = e^(*q*_*a*_−*q*_*a* + 1_)^ must be interpreted to mean $\left(m_{0}\ell_{0}^{2}/t_{0}^{2}\right){\text{e}}^{(q_{a}-q_{a+1})/\ell_{0}}$, and the factor $\frac{1}{2}$ in the dimensionless kinetic energy must be interpreted to mean $\frac{1}{2}m_{0}^{-1}$. In “units where *t*_0_ = *ℓ*_0_ = *m*_0_ = 1”. Consider now a change of reference units to

$$\begin{array}{c} ({\tilde{t}}_{0},{\tilde{\ell }}_{0},{\tilde{m}}_{0})=(t_{0}/\lambda_{t},\ell_{0}/\lambda_{\ell },m_{0}/\lambda_{m}), \end{array}$$ (20)

with all physical quantities fixed. It is rather obvious that dimensionless coordinates will change as

$$\begin{array}{c} t\to \tilde{t}=\lambda_{t}t, \end{array}$$ (21a)

$$\begin{array}{c} q\to \tilde{q}=\lambda_{q}q, \end{array}$$ (21b)

$$\begin{array}{c} p\to \tilde{p}=(\lambda_{m}\lambda_{\ell }/\lambda_{t})p. \end{array}$$ (21c)

In this context, the statement *t* → λ_*t*_ *t* is shorthand for i) a change of reference units $t_{0}\to {\tilde{t}}_{0}=t_{0}/\lambda_{t}$, implying ii) the transformation of Eq (21a), often followed by iii) a symbol renaming back to the original one, $\tilde{t}\to t$.

The corresponding transformations of *V*(*q*) and $\frac{1}{2}p^{2}$ are less obvious,

$$\begin{array}{c} V(q)\to \tilde{V}\left(\tilde{q}\right)=\left(\lambda_{m}\lambda_{\ell }^{2}/\lambda_{t}^{2}\right)\;V\left(\lambda_{q}\tilde{q}\right), \end{array}$$ (21d)

$$\begin{array}{c} \frac{1}{2}p^{2}\to \frac{1}{2}\lambda_{m}^{-1}\;{\tilde{p}}^{2}, \end{array}$$ (21e)

and cannot be deduced from the dimensionless expressions without knowledge of which physical quantity they represent (energy in this case).

## C Code snippets

In this section we provide some information of how the routines in the HOMsPY package can be used to create the symplectic solvers for the Toda lattice Hamiltonian, and how these solvers can be used to solve an initial value problem from provided initial data.

The package itself can downloaded from the CPC Program Library at http://cpc.cs.qub.ac.uk/summaries/ADTV, by providing the Catalogue Id AESD v1.0. The code, with accompanying information which need not be repeated here, is found by unpacking the downloaded .tar-file. Since the package is written Python 2.7, we provide code snippets illustrating how the solvers can be accessed by Python 3 code.

One way to continue is to add the code in Listing 1 to the examples/makeExamples.py file. But for our project we made a new folder TodaLattice, and added it to a new file makeTodaLattices.py.

**Listing 1**. **Constructing KiMoKi solvers for a Toda lattice model**

def makeTodaLattices():

 # *Choose names for coordinates and momenta*

 q1, q2, p1, p2 = sympy.symbols([’q1’, ’q2’, ’p1’, ’p2’])

 qvars = [q1, q2]; pvars = [p1, p2]

 # *Define potential in terms of coordinates*

 V = (exp(2*q2+2*sqrt(3)*q1) + exp(2*q2-2*sqrt(3)*q1) + exp(-4*q2) − 3)/24

 kimoki.makeModules(’TodaLattices’, V, qvars, pvars, DP = True, MP = True, MAXORDER = 8, VERBOSE = True)

By running this code several files will be created. The most important one is TodaLattices.py, which contains the *KiMoKi* solvers up to 8th order. This file should *not* be modified manually; is not intended to be studied in detail by humans. But (in particular) the multiprecision code should be checked against unintended conversions to floating point numbers. F.i., if the final division /24 in the above definition of V is replaced by a pre-multiplication (1/24)*, then this factor will be converted to a double precision number at an early stage, and thereby pollute all multiprecision accuracy.

The file runTodaLattices.py provide some usage examples, intended to be modified and extended. Since this file will be overwritten the next time makeTodaLattices is executed, it is recommended to work on a renamed copy.

The current version of the *HOMsPY* package, with all its output, is written in Python 2.7. But functions can be accessed from Python 3 through an interface like the one in Listing 2. Data exchange via pickle files may seem primitive, but this has the advantage of documenting (preserving) the arguments and data being used.

**Listing 2**. **Python 3 code calling Python 2.7 function**.

def get_ivpsoln(**kwargs):

 ″″″*Solve an initial value problem by use of KiMoKi*.

 *The KiMoKi solver routines are currently written in Python2.7. This is a simple Python 3 interface using ’subprocess.call()’. Arguments and results are communicated via pickle files, for which some care with protocol and encoding is required*.″″″

 # *Default arguments*:

 args = {’argfile’: ’ivpargs’, ’soln’: ’ivpsoln’, ’tau’: 0.05, ’tmax’: 50., ’order’: 4, ’z0’: (0., 0., 5., 5.)}

 args = {**args, **kwargs} # *Override defaults*

 if not os.path.isfile(f″./{args[’soln’]}.pkl″):

  with open(f″{args[’argfile’]}.pkl″, ’wb’) as outfile:

   pickle.dump(args, outfile, protocol = 2)

  subprocess.call([″python2″, ″./run todalattices.py″, ″solve_ivp″, args[’argfile’]])

 with open(f″{args[’soln’]}.pkl″, ’rb’) as infile:

  soln = pickle.load(infile, encoding=’latin1’)

 return soln

On the Python 2.7 side we copied runTodaLattices.py to run_todalattices.py. All existing functions except computeSolution(…) can be deleted, and the function in Listing 3 must be added.

**Listing 3**. **Python 2.7 function called from Python 3**.

def solve_ivp(argfile):

 ″″″*Interface to python3 code through a subprocess call*.

 *Calling arguments (’args’), and the returned solution (’zt’) are communicated through pickle files*.″″″

 # *All arguments with default values*

 args = {’tmax’: 50., ’tau’: 0.05, ’order’: 4, ’z0’: (0., 0., 5., 5.), ’soln’: ″ivpsoln″}

 with open(argfile, ’rb’) as infile:

  kwargs = pickle.load(infile)

 for key, value in kwargs.items():

  args[key] = value # *Override defaults*

 tmax, tau = args[’tmax’], args[’tau’]

 nMax = 1+int(tmax/tau)

 z0 = numpy.array(args[’z0’])

 zt = computeSolution(z0, tau, args[’order’], nMax)

 with open(″%s.pkl″ % args[′soln′], ′wb′) as outfile:

  pickle.dump(zt, outfile)

Further, the last two blocks in run_todalattices.py were changed to the one of Listing 4.

**Listing 4**. **Python 2.7 main entry point**

funcs = {’solve_ivp’: solve_ivp, ’find_sections’: find_sections

if __name__ == ″__main__″:

 ″″″*Execute the function named by sys.argv[1], with argument sys.argv[2]*.

 ″″″

 argc = len(sys.argv)

 func = sys.argv[1] if argc > 1 else ″solve_ivp″

 argfile = ″%s.pkl″ % sys.argv[2] if argc > 2 else ″args.pkl″

 funcs[func](argfile)

## D Explicit expressions

On request from a reviewer we here for convenience include the explicit expressions used in the algorithms of Eq (15). The rest of this section is an essentially unedited copy of a section with the same name, previously published by Mushtaq and Olaussen [8]:

Explicit (but compact) expressions for the terms of order *τ*^2^, *τ*^4^, and *τ*^6^ were given in [5, 19]. With the notation,

$$\begin{array}{cc} & \partial_{a}\equiv \frac{\partial }{\partial q^{a}},\;\partial^{a}\equiv M^{ab}\partial_{b},\;p^{a}\equiv M^{ab}p_{b},\;D\equiv p_{a}\partial^{a},\;\bar{D}\equiv \left(\partial_{a}V\right)\partial^{a}, \end{array}$$

where the *Einstein summation convention is employed* (an index which occur twice, once in lower position and once in upper position, are implicitly summed over all available values; i.e, *M*^*ab*^∂_*b*_ ≡ ∑_*b*_ *M*^*ab*^∂_*b*_—we generally use the matrix *M* to rise an index from lower to upper position), they are

$$\begin{array}{c} T_{2}=-\frac{1}{12}D^{2}V\tau^{2}, \end{array}$$ (22a)

$$\begin{array}{c} T_{4}=\frac{1}{720}(D^{4}-9\bar{D}D^{2}+3D\bar{D})V\tau^{4}, \end{array}$$ (22b)

$$\begin{array}{c} T_{6}=-\frac{1}{60480}(2\;D^{6}-40\;\bar{D}D^{4}+46\;D\bar{D}D^{3}-15\;D^{2}\bar{D}D^{2}\\ \;+54\;{\bar{D}}^{2}D^{2}-9\;\bar{D}D\bar{D}D-42\;D{\bar{D}}^{2}D+12\;D^{2}{\bar{D}}^{2})V\tau^{6} \end{array}$$ (22c)

$$\begin{array}{c} V_{2}=\frac{1}{24}\bar{D}V\tau^{2}, \end{array}$$ (22d)

$$\begin{array}{c} V_{4}=\frac{1}{480}{\bar{D}}^{2}V\tau^{4}, \end{array}$$ (22e)

$$\begin{array}{c} V_{6}=\frac{1}{161280}(17\;{\bar{D}}^{3}-10\;{\bar{D}}_{3})V\tau^{6}. \end{array}$$ (22f)

In this last line we have introduced

$$\begin{array}{c} {\bar{D}}_{3}\equiv \left(\partial_{a}V\right)\left(\partial_{b}V\right)\left(\partial_{c}V\right)\partial^{a}\partial^{b}\partial^{c}. \end{array}$$ (23)

The *kick*-steps can still be integrated directly, since the *V*_2*k*_’s only depend on ***q***. However, the *T*_2*k*_’s (for *k* ≥ 1) in general depend on both ***q*** and ***p***; hence the *move*-steps cannot be integrated directly. To overcome this problem we introduce a generating function

$$\begin{array}{c} G\left(q,P;\tau \right)=\sum_{0\leq k\leq N}G_{k}\left(q,P\right)\;\tau^{k} \end{array}$$ (24)

such that the transformation (***q***, ***p***) → (***Q***, ***P***) defined implicitly by

$$\begin{array}{c} Q^{a}=\frac{\partial G}{\partial P_{a}},\;p_{a}=\frac{\partial G}{\partial q^{a}}, \end{array}$$ (25)

preserves the symplectic structure exactly, and reproduce the time evolution generated by *T*_eff_ to order *τ*^*N*^. Here *Q*^*a*^ is shorthand for *q*^*a*^(*t* + *τ*), and *P*_*a*_ shorthand for *p*_*a*_(*t* + *τ*). The explicit expressions for the coefficients $G_{k}$ are

$$\begin{array}{c} G_{0}=q^{a}P_{a}, \end{array}$$ (26a)

$$\begin{array}{c} G_{1}=\frac{1}{2}P^{a}P_{a}, \end{array}$$ (26b)

$$\begin{array}{c} G_{2}=0, \end{array}$$ (26c)

$$\begin{array}{c} G_{3}=-\frac{1}{12}D^{2}V, \end{array}$$ (26d)

$$\begin{array}{c} G_{4}=-\frac{1}{24}D^{3}V, \end{array}$$ (26e)

$$\begin{array}{c} G_{5}=-\frac{1}{240}(3\;D^{4}+3\;\bar{D}D^{2}-D\bar{D}D)V, \end{array}$$ (26f)

$$\begin{array}{c} G_{6}=-\frac{1}{720}({2\;D}^{5}+8\;\bar{D}D^{3}-5\;D\bar{D}D^{2})V, \end{array}$$ (26g)

$$\begin{array}{c} G_{7}=-\frac{1}{20160}({10\;D}^{6}+10\;\bar{D}D^{4}+90\;D\bar{D}D^{3}-75\;D^{2}\bar{D}D^{2}\\ \;+\;18\;{\bar{D}}^{2}D^{2}-3\;\bar{D}D\bar{D}D-14\;D{\bar{D}}^{2}D+4\;D^{2}{\bar{D}}^{2})V, \end{array}$$ (26h)

$$\begin{array}{c} G_{8}=-\frac{1}{40320}(3\;D^{7}-87\;\bar{D}D^{5}+231\;D\bar{D}D^{4}-133\;D^{2}\bar{D}D^{3}+63\;{\bar{D}}^{2}D^{3}\\ \;-3\;D{\bar{D}}^{2}D^{2}-21\;D^{2}{\bar{D}}^{2}D+\;4\;D^{3}{\bar{D}}^{2}-63\;\bar{D}D\bar{D}D^{2}\\ \;+25\;D\bar{D}D\bar{D}D)V. \end{array}$$ (26i)

The Eqs (22,26) define the *kick-move-kick* scheme for a general potential *V*. If one uses all the listed terms the local error becomes of order *τ*^9^, and the scheme will respect long-time conservation of energy to order *τ*^8^.
